# INVESTIGATING VARIANTS OF TUMOR SUPPRESSOR GENES IN HISTIOCYTIC SARCOMA

**DOI:** 10.51894/001c.123027

**Published:** 2024-08-30

**Authors:** Yutong Liang

**Affiliations:** 1 College of Osteopathic Medicine Michigan State University

## 57

### INTRODUCTION

Tumor suppressor genes play key roles in many cancer processes. They control functions such as the cell cycle and maintaining DNA integrity. Histiocytic sarcoma (HS) is a rare disease seen in both humans and canines with inherited predisposition likely involving the inactivation of tumor suppressor genes. Histiocytes normally can be induced by cytokines to become tissue macrophages and dendritic cells. The prevalence of HS in certain breeds of canines is higher, such as the Bernese Mountain dog, but a predisposing factor in humans has not yet been identified. However, it is known that many human syndromes are also due to inherited mutations in tumor suppression genes following the “two-hit” hypothesis.

### OBJECTIVE

To analyze if variants in various tumor suppressor genes contribute to the high incidence of Histiocytic Sarcoma in Bernese Mountain dogs.

### METHODS

Studies of variant allele frequencies will be conducted using a series of DNA samples from a set of HS canine populations utilizing both constitutive and tumor cell line DNA. Twenty previously identified tumor suppressor genes are selected based on whole genome sequencing (WGS) analysis of HS cell lines and observed to have coding variants predicted by software SIFT to have moderate to severe impact on tumor suppressor protein function These genes will be further analyzed to determine their potential in contributing to tumorigenesis in HS. Allele frequencies will be determined using genotyping or Sanger sequencing.

### RESULTS

The identified tumor suppressor genes are anticipated to display loss of heterozygosity. Variant frequencies will then be assessed in the general canine population via exploring variant databases or experimentally determined using DNA from affected and non-affected canine DNA available in the lab.

### CONCLUSION

Based on the identified variants and projected results, the identified tumor suppressor genes are expected to display loss of heterozygosity and involve various degrees of disruption to gene function. Orthogonal studies to probe effects on downstream pathways may be conducted to further evaluate impact of the variants using the canine HS cell lines. The ultimate goal of the project is to delineate key pathways disrupted that give rise to HS malignancy.

